# The relationships between paediatric nurses' social support, job satisfaction and patient adverse events

**DOI:** 10.1002/nop2.907

**Published:** 2021-05-02

**Authors:** Haitham Khatatbeh, Tariq Al‐Dwaikat, András Oláh, David Onchonga, Sahar Hammoud, Faten Amer, Viktória Prémusz, Annamária Pakai

**Affiliations:** ^1^ Doctoral School of Health Sciences Faculty of Health Sciences University of Pécs Pécs Hungary; ^2^ Department of Community and Mental Health Faculty of Nursing Jordan University of Science and Technology Irbid Jordan; ^3^ Institute of Nursing Sciences, Basic Health Sciences and Health Visiting Faculty of Health Sciences University of Pécs Pécs Hungary

**Keywords:** job satisfaction, manager support, medication error, nosocomial infection, paediatric nurse, patient falls, pressure ulcer

## Abstract

**Aims:**

To explore the relationships of family, co‐worker and manager support with paediatric nurses' satisfaction and their perception of adverse events. Furthermore, this study aimed to assess the job satisfaction, social support and the perceived patient adverse events.

**Design:**

This study used a cross‐sectional correlational design.

**Methods:**

A convenient sample of 225 paediatric nurses was selected from nine hospitals in Jordan. Both the Pearson correlations and multiple regression tests were used in the analysis. The study was prepared and is reported according to the STROBE checklist.

**Results:**

Significant and positive correlations were found between paediatric nurses' job satisfaction and the social support they receive. Significant negative correlations were also found between adverse events and both family and manager support. The multiple regression results showed that manager support is a significant negative predictor of both pressure ulcers and patient falls, and family support significantly predicted paediatric nurses' job satisfaction.

## 1 INTRODUCTION

Adverse events are those avoidable outcomes that result from wrong healthcare services, not from the disease itself (Van den Bos et al., 2011). Adverse events are common in all healthcare systems and are considered an important aspect of patient safety. Only in the United States, around 440,000 people died in 2013 because of avoidable adverse events (Schwendimann et al., 2018). In 2008, the adverse events' estimated cost was approximately 17 billion dollars in the United States (Van den Bos et al., 2011).

The causes of the adverse events are numerous and interrelated with job satisfaction and support the nurses receive. For instance, work‐related stress was associated with more adverse events (Karimi et al., 2018). On the other hand, recognition of nurses, which is considered a type of support, was associated with higher job satisfaction (Al Maqbali, 2015).

A Jordanian study found that the most common types of nurse‐perceived patient adverse events are medication errors, nosocomial infections, pressure ulcers and patient falls (Hayajneh et al., 2010). In the same study, researchers estimated the adverse events occurred to 28% of the admitted patients to Jordanian hospitals (Hayajneh et al., 2010). Although the study of Hayajneh et al. (2010) has a small sample size, the Internet‐based data collection supports the results' generalizability by representing the different healthcare sectors in Jordan (military, governmental, university‐affiliated and private hospitals).

A recent Jordanian study showed that the mean number of medication errors committed by Jordanian nurses over their career was found to be 28.3 (*SD* = 16.6) (Alrabadi et al., 2020). The demographic characteristics that had significant relationships with the number of committed medication errors were the experience and type of hospital (Alrabadi et al., 2020). In their study, Alrabadi et al. (2020) assessed only nurses' perceptions instead of reviewing incident reporting charts. However, incident reporting charts do not necessarily reflect the situation as fear of punitive actions prevents the nurses from reporting the medication errors.

Regarding the nosocomial infections, a study found that 48 out of 507 inpatients at neonatal intensive care units (NICU) developed at least one nosocomial infections (Rangelova et al., 2020). The study of Rangelova et al. (2020) collected data only from NICU's which underrepresent the other paediatric patients. However, the relatively large sample size makes the results more generalizable. Additionally, a retrospective study conducted in Jordan revealed that surgical site infection's prevalence is 5.4% (Al‐Awaysheh, 2018). While the study of Al‐Awaysheh (2018) collected data from one hospital, it depended on the hospital records, ensuring the results' validity.

Several studies on patient falls' incidences and correlations were conducted. A study found that the average rate of patient falls among admitted patients was 3.82 (*SD* = 2.74), and it was negatively correlated with nurses' staffing levels (Kalisch et al., 2012). Similarly, another study found that both small hospitals and less staffing levels resulted in more patient falls (Dunton et al., 2004). Although the study of Dunton et al. (2004) used secondary data, it assesses 282 healthcare institutions, which supports the results' validity.

Job satisfaction and patient safety might be correlated. In other words, improved nurses' job satisfaction was associated with less frequent patient adverse events (Boamah et al., 2018). The literature has shown that nurses have relatively low to moderate job satisfaction levels (Al Maqbali, 2015; Aljohani, 2019). A survey of 2,418 nurses in Saudi Arabia showed low to moderate job satisfaction levels (Aljohani, 2019). The large sample size and multi‐site setting of this study (Aljohani, 2019), support the results' validity. Another study showed moderate job satisfaction levels among hospital nurses in Oman (Al Maqbali, 2015). However, the relatively low sample size selected by Al Maqbali (2015) from one hospital might constrain the results' generalizability.

So, it is necessary to understand the variables that contribute to nurses' job satisfaction. A study demonstrated that educational level significantly predicted the level of job satisfaction (Aljohani, 2019). Also, the age and shift work were significantly affecting job satisfaction levels (Al Maqbali, 2015). Jordanian nurses working at the ministry of health (MOH) hospitals showed higher job satisfaction levels than those working at private hospitals (Abdelhafiz et al., 2016). A significant positive relationship was found between the leadership style and level of job satisfaction among Jordanian nurses (Abdelhafiz et al., 2016).

Social support is essential in improving patient safety even among clinical nursing students (Li et al., 2021). Marital status, nursing model and organizational structure were the most common predictors of social support among nurses (Amarneh, 2017). This multi‐site study (Amarneh, 2017) used data from 13 hospitals, which make its results generalizable to Jordanian nurses. Both manager and co‐workers' support are essential factors for coping when nurses care for dying patients (Chang, 2018). Three types of social support were studied in relation to nurses' self‐efficacy, namely co‐workers, family and friends' support (Wang et al., 2018). Co‐workers' support was correlated with the nurses' self‐efficacy and managing the hard times (Wang et al., 2018). The structural equation modelling used by Wang et al. (2018) based on a theoretical model, in addition to the relatively large sample size makes the findings more valid and generalizable.

Nursing studies regarding job satisfaction, social support and adverse events are many. However, few studies discussed the relationships between these concepts. To the best of our knowledge, no studies have discussed the impact of family support, co‐workers support and manager support on the patient adverse events and nurses' job satisfaction in Jordan. This study therefore aimed to (1) assess the paediatric nurses' job satisfaction, social support, the perceived patient adverse events and (2) explore the relationships of social support with both paediatric nurses' satisfaction and their perception of adverse events. While we hypothesize that a relationship exists between paediatric nurses' job satisfaction and the social support they get, we also assume that social support and patient adverse events are somehow correlated.

## 2 METHODOLOGY

### 2.1 Design and sample

A cross‐sectional, correlational design was used to explore the relationships between social support, job satisfaction and patient adverse events. The researchers selected eight MOH hospitals and one university‐affiliated hospital to represent MOH and university‐affiliated hospitals in Jordan. It is well‐known that most people live in Jordan's northern and central regions (Department of Jordanian Statistics, 2018). To reduce sampling bias, eight hospitals from the northern and central areas, and only one hospital from the southern area were selected. Out of 300 paediatric nurses who met the inclusion criteria, the number of paediatric nurses who responded to the study questionnaires was 225. The inclusion criteria were being a Jordanian staff nurse, holding at least a 2‐year nursing diploma and working for at least 1 year in a paediatric unit/ward. A pilot study was conducted on 35 nurses to assess the ease of instrument implementation and any validity concerns, and no issues were found. The study was prepared and is reported according to the STrengthening the Reporting of OBservational studies in Epidemiology (STROBE) checklist (Von Elm et al., 2014). See STROBE checklist in Appendix S1.

### 2.2 Sample size calculation

The G*Power software was used in the sample size calculation to ensure adequate statistical power (G*Power, 2020). Using the multiple regression approach, significance set at 1%, power set at 0.99, effect size of 0.15 and the number of predictors being three; a total sample size of 205 subjects was required. In the post hoc analysis, the sample of 225 participants provided a power of 0.995, which is statistically enough to make conclusions.

### 2.3 Measures

Demographic variables assessed in this study were gender, marital status, educational level, hospital type, unit/ward, age and experience (years).

This study assessed four types of nurse‐perceived patient adverse events: medication errors, pressure ulcers, patient falls and nosocomial infections. Like a previous study (Van Bogaert et al., 2014), nurses in this study were asked how frequently the patient adverse events occurred. Four items of 6‐point Likert scales were used (never, once a month or less, a few times a month, once a week, a few times a week, every day). To have more discrete choices, the four items were re‐coded later in the analysis phase into 4 points (never, monthly, weekly, daily). The Cronbach's alpha for the four patient adverse events was 0.833, which showed good internal consistency. This is similar to the Cronbach's alpha (*α* > .80) found by a previous study (Van Bogaert et al., 2014).

Using three items of 5‐point Likert scales, nurses were asked about the social support from their families, co‐workers and managers (Very weak, weak, fair, good, very good). The Cronbach's alpha for these three questions was satisfactory (*α* = .74). Nurses were also asked directly about their job satisfaction using a 5‐point Likert item (Very poor, poor, intermediate, good, very good).

### 2.4 Data collection

After the necessary research ethics committee approvals were obtained, data collection took place in the selected nine hospitals between December 2019–March 2020. The printed questionnaires were handed to the head nurses of paediatric units/wards in sealed envelopes. In the same way, the answered questionnaires were collected back on the next day. The cover sheet attached to each questionnaire explained the research aims and a consent form to participate in the study. The anonymous, voluntary participation and the right to withdraw from the study were assured in the consent form.

### 2.5 Data analysis

Statistical Package for Social Sciences (SPSS), version 20.0, was used in this research with a level of significance set as *p* < .05. Descriptives and frequencies were used to describe the characteristics of participants. In terms of data normal distribution, the Kolmogorov–Smirnov test was done for the four types of patient adverse events and job satisfaction. The Kolmogorov–Smirnov test results were all significant; this means that the data are significantly different from the normal distribution. However, the researchers preferred to use the parametric tests for many reasons. First, parametric tests are more powerful than the non‐parametric tests. Second, the results of Kolmogorov–Smirnov test are not necessarily true especially with the relatively large sample size (Steinskog et al., 2007). Last, according to kurtosis, skewness values and histograms, the data were normally distributed. The Pearson correlation test was also used to find the correlations between the studied variables. Multiple regression was used to assess whether social support predicts the four types of adverse events and job satisfaction.

### 2.6 Ethical considerations

Ethical permissions were obtained from the central institutional review boards at the MOH in Jordan and a university‐affiliated hospital. Each participant was asked to sign an informed consent form on the cover sheet of questionnaires.

## 3 RESULTS

### 3.1 Participants characteristics

The mean age of the participants (*N* = 225) was 33.6 years (*SD* = 6.50), with a mean experience of 11.1 years (*SD* = 6.74). Most of the participants were females 94.2% (*N* = 212) and married 82.7% (*N* = 186). Regarding the educational level, the majority of nurses were bachelor's degree holders 87.6% (*N* = 197). About 52.7% (*N* = 117) of the nurses were assigned either in paediatric or neonatal intensive care units. Concerning the type of hospital, approximately 70.2% (*N* = 158) worked at MOH hospitals while the rest work at the university‐affiliated hospital (Table 1).

**TABLE 1:** Participants characteristics

Variable	*N*	Percentage
Gender		
Male	11	4.9
Female	212	94.2
Missing	2	0.90
Marital status		
Single	34	15.1
Married	186	82.7
Divorced	4	1.8
Widowed	1	0.40
Education		
Diploma	5	2.2
Bachelor's degree	197	87.6
Master's degree	23	10.2
Hospital		
Governmental	158	70.2
University‐affiliated	67	29.8
Unit/Ward		
Paediatric ward	70	31.5
Paediatric ER	12	5.4
PICU	42	18.9
NICU	75	33.8
Paediatric oncology	6	2.7
Other paediatric ward/unit	17	7.7

	M	*SD*
Age (years)	33.60	6.50
Experience (years)	11.1	6.74
Job satisfaction	2.93	1.09
Family support	3.56	1.16
Co‐worker support	3.28	0.99
Manager support	3.42	1.06
Medication errors	1.46	0.68
Pressure ulcers	1.44	0.62
Patient falls	1.46	0.60
Nosocomial infection	1.75	0.71

### 3.2 Job satisfaction, social support and adverse events

The mean scores for job satisfaction, family support, co‐worker support and manager support were 2.93 (*SD* = 1.09), 3.56 (*SD* = 1.16), 3.28 (*SD* = 0.99), and 3.42 (*SD* = 1.06), respectively. The overall mean score for the three types of support was 3.42. The mean scores for the medication errors, pressure ulcers, patient falls and nosocomial infections were 1.46 (*SD* = 0.68), 1.44 (*SD* = 0.62), 1.46 (*SD* = 0.60) and 1.75 (*SD* = 0.71), respectively. The overall mean score for the four types of adverse events was 1.53 (Table 1).

### 3.3 Pearson correlations

Table 2 shows the significant negative correlations found between nurses' age and the perceived frequency of both medication errors (*r* = −.19, *p* < .01) and pressure ulcers (*r* = −.11, *p* < .05). No significant correlations were found between nurses' gender nor marital status with their job satisfaction or adverse events. Both nurses' educational level and type of the hospital were significantly correlated with the perceived frequency of nosocomial infections (*r* = −.17, *p* < .01) and (*r* = .16, *p* < .05), respectively. Unit/Ward the nurses work in was significantly correlated with family support (*r* = .12, *p* < .05), co‐worker support (*r* = .18, *p* < .01), job satisfaction (*r* = .12, *p* < .05), patient falls (*r* = −.15, *p* < .05) and nosocomial infections (*r* = −.14, *p* < .05).

**TABLE 2:** Correlations among study variables (*N* = 225)

	(1)	(2)	(3)	(4)	(5)	(6)	(7)	(8)	(9)	(10)	(11)	(12)	(13)	(14)
(1) Age	1	−0.10	0.23**	−0.18**	0.06	0.40**	−0.03	0.11	−0.08	0.09	−0.19**	−0.11*	−0.08	−0.01
(2) Gender		1	−0.02	0.05	−0.05	−0.01	−0.01	−0.10	−0.07	−0.02	0.06	−0.01	0.07	−0.08
(3) Marital status			1	−0.27**	0.05	0.09	−0.01	0.03	−0.03	0.05	−0.02	0.06	−0.06	−0.05
(4) Educational level				1	0.09	−0.19**	0.07	0.05	0.03	−0.03	−0.06	−0.10	−0.09	−0.17**
(5) Unit/Ward					1	−0.19**	0.12*	0.18**	0.10	0.12*	−0.05	−0.11	−0.15*	−0.14*
(6) Type of hospital						1	−0.12*	0.04	−0.06	0.04	−0.09	−0.03	−0.02	0.16*
(7) Family support							1	0.40**	0.53**	0.24**	−0.14*	−0.14*	−0.13*	−0.14*
(8) Co‐worker support								1	0.53**	0.13*	−0.07	−0.08	−0.09	−0.11
(9) Manager support									1	0.23**	−0.18**	−0.23**	−0.21**	−0.15*
(10) Job satisfaction										1	−0.10	−0.14*	−0.08	−0.08
(11) Medication errors											1	0.62**	0.59**	0.47**
(12) Pressure ulcers												1	0.50**	0.49**
(13) Patient falls													1	0.46**
(14) Nosocomial infections														1

* Correlation is significant at the 0.05 level (1‐tailed).

** Correlation is significant at the 0.01 level (1‐tailed).

Nurses' job satisfaction was negatively correlated with the perceived frequency of pressure ulcers (*r* = −.14, *p* < .05). Job satisfaction was positively correlated with the three types of support; family support (*r* = .24, *p* < .01), co‐worker support (*r* = .13, *p* < .05) and manager support (*r* = .23, *p* < .01). The co‐workers' support was not significantly correlated with perceived frequencies of the four adverse events. The family support was negatively correlated with perceived frequencies of medication errors (*r* = −.14, *p* < .05), pressure ulcers (*r* = −.14, *p* < .05), patient falls (*r* = −.13, *p* < .05) and nosocomial infections (*r* = −.14, *p* < .05). Manager support was also negatively correlated with perceived frequencies of medication errors (*r* = −.18, *p* < .01), pressure ulcers (*r* = −.23, *p* < .01), patient falls (*r* = −.21, *p* < .01) and nosocomial infections (*r* = −.15, *p* < .05).

### 3.4 Regression analysis

In order to find the significant model predicting nurses' perceived frequencies of adverse events and job satisfaction, multiple regression analyses (Table 3) were done. The predicting variables for the five models were family support, co‐worker support and manager support. These variables all together significantly predicted medication errors (*F* (3, 221) = 2.694, *p* = .047, $R_{\text{Adjusted}}^{2}$ = .022), pressure ulcers (*F* (3, 221) = 4.374, *p* = .005, $R_{\text{Adjusted}}^{2}$ = .043), patient falls (*F* (3, 221) = 3.450, *p* = .017, $R_{\text{Adjusted}}^{2}$ = .032) and job satisfaction (*F* (3, 221) = 5.896, *p* = .001, $R_{\text{Adjusted}}^{2}$ = .062). However, these variables did not predict the nosocomial infections (*F* (3, 221) = 2.041, *p* = .109, $R_{\text{Adjusted}}^{2}$ = .014). Within the models predicting medication errors and nosocomial infections, no significant predictors were found. Manager support was significantly predicting both pressure ulcers (*β* = −.246, *p* < .01) and patient falls (*β* = −.213, *p* < .05). Regarding the job satisfaction model, family support was the significant predictor (*β* = .17, *p* < .05).

**TABLE 3:** Regression results for job satisfaction and nurse‐perceived patient adverse events (*N* = 225)

Dependent variable	AE1 (medication errors)		AE2 (pressure ulcers)		AE3 (patient falls)		AE4 (nosocomial infections)		Job satisfaction	
Model summary	*F* (3, 221) = 2.694, *p* = .047, $R_{\text{Adjusted}}^{2}$ = .022		*F* (3, 221) = 4.374, *p* = .005, $R_{\text{Adjusted}}^{2}$ = .043		*F* (3, 221) = 3.450, *p* = .017, $R_{\text{Adjusted}}^{2}$ = .032		*F* (3, 221) = 2.041, *p* = .109, $R_{\text{Adjusted}}^{2}$ = .014		*F* (3, 221) = 5.896, *p* = .001, $R_{\text{Adjusted}}^{2}$ = .062	
Predictors	*β*	*t*	*β*	*t*	*β*	*t*	*β*	*t*	*β*	*t*
Family support	−.075	−0.952	−.029	−0.368	−.026	−0.330	−.080	−1.012	.170	2.200*
Co‐worker support	.039	0.491	.058	0.741	.035	0.442	−.032	−0.402	−.011	−0.137
Manager support	−.156	−1.830	−.246	−2.921**	−.213	−2.515*	−.086	−1.001	.147	1.758

* *p* < .05.

** *p* < .01.

## 4 DISCUSSION

The paediatric nurses' family support, co‐worker support, manager support, job satisfaction and perceived adverse events were explored in this study. A mean score of 2.93, on five‐point satisfaction scale, reflects a relatively low job satisfaction among paediatric nurses. The low levels of nurses' job satisfaction in this study are congruent with two previous studies (Al Maqbali, 2015; Aljohani, 2019). The relatively average social support among Jordanian nurses was interpreted by the overall mean score of 3.42 on five‐point scale. This finding regarding the social support is consistent with a previous study in Jordan (Amarneh, 2017). The overall mean score of 1.53 means a low rate of adverse events as perceived by paediatric nurses. The relatively low perceptions of adverse events might reveal the fear of nurses from punishment or disciplinary actions if they declare high rate of adverse events. This fear was clearly discussed in a recent systematic review (Al‐zubi et al., 2020).

In the present study, we also explored the relationships of family, co‐worker and manager support with paediatric nurses' satisfaction and their perceived patient adverse events. The Pearson correlation results found a significant negative correlation between nurses' age and the perceived frequency of medication errors and pressure ulcers. The older nurses perceive less frequent medication errors and pressure ulcers. This finding might be related to the more experience the nurses will have and the more knowledge of patient safety importance. Results also showed a significant negative correlation between nurses' educational level and nosocomial infections. The nurses with higher educational levels will perceive less frequent nosocomial infections. This finding can be explained since nurses with higher educational degrees have higher awareness on infection control measures that aim at reducing the transmission of nosocomial infections (Hammoud et al., 2017). A significant correlation was also found between the type of hospital and nosocomial infections. The nurses working at MOH hospitals perceived more frequent nosocomial infections than nurses working at university‐affiliated hospitals. This finding sounds logical because university hospitals apply more strict policies on IC and quality of care. This is consistent with the results of Hammoud et al. (2017), where private hospitals were found to engage their patients more than public hospitals in IC. Patient engagement in IC was lately recommended as a way to reduce the spread of nosocomial infections (Hammoud et al., 2020).

A significant negative relationship was found between nurses' job satisfaction and the perceived frequency of pressure ulcers. That is, the improved nurses' job satisfaction causes less frequent patient adverse events. This result matches what was found in previous studies (Boamah et al., 2018; Karimi et al., 2018), which revealed that less work stress and more job satisfaction leads to less patient adverse events.

Positive correlations were found between job satisfaction and the three types of support the nurses get. The stronger support from family, co‐workers and managers the higher the job satisfaction. This finding is congruent with a previous study, which found a significant relationship between nurses' job satisfaction and leadership styles such as laissez‐faire or democratic styles as it encompasses more support than other styles (Abdelhafiz et al., 2016). This result is also congruent with what a previous study found that nurses' recognition is correlated with more job satisfaction (Al Maqbali, 2015).

Negative correlations were also found between both family and manager support with the four types of adverse events. The higher family and manager support for paediatric nurses, the less frequent medication errors, pressure ulcers, patient falls and nosocomial infections will be perceived. This result matches the findings found by a previous study that manager support contributes to paediatric patient safety (Khatatbeh et al., 2020). This finding is also supported by another study which concluded that patient safety could be enhanced by creating a healthy work environment for nurses (Amarneh, 2017).

Multiple regression results showed that manager support was a significant predictor of both pressure ulcers and patient falls. In other words, the better work environment is accompanied with less adverse events which matches the results found previously (Cho et al., 2016). This finding is also supported by a previous study, which found that work stress and adverse events are associated (Karimi et al., 2018). In terms of job satisfaction, multiple regression also showed that family support is a significant predictor. This result means that paediatric nurses' job satisfaction will be higher when they get more family support. This result matches what it was previously found that social support contributes to revolving the workplace into a healthy work environment (Amarneh, 2017).

### 4.1 Limitations

This study is limited by some factors, such as the convenient sample selected. Using nurses' perceptions instead of medical records and incidence reports to assess patient adverse events is another limitation. So, we recommend future researchers to use patients records and documented incidence reports to collect adverse events data. Asking nurses directly about their job satisfaction and the support they receive might be another limitation. Future researcher might consider using more structured questionnaires in assessing job satisfaction and support. However, we believe that our results can be generalized, because our sample covered all areas in Jordan.

## 5 CONCLUSIONS

The present study revealed significant negative correlations between both family and manager support and nurses perceived adverse events. Furthermore, correlations were found between adverse events and the demographic characteristics. For example, a significant negative correlation was found between age and both medication errors and pressure ulcers. Unit/Ward was also significantly and negatively correlated with both patient falls and nosocomial infections. The results also discovered that family support is a positive predictor for job satisfaction and that manager support is a negative predictor for both pressure ulcer and patient falls.

## 6 IMPLICATIONS

This study provides vital information for the nursing society, especially paediatric nurses' managers and families. Families of paediatric nurses should be aware that their support is essential to enhance their relative nurses' satisfaction and the safety of paediatric patients. It is also essential that nursing managers understand how to boost co‐workers and manager support to improve paediatric nurses' satisfaction, decreasing patient adverse events and improving patient safety.

## CONFLICT OF INTEREST

The authors declare no conflict of interest.

## AUTHOR CONTRIBUTION

All authors are responsible for the reported research and have approved the manuscript as submitted. All authors have contributed in the manuscript: Haitham Khatatbeh, Tariq Al‐Dwaikat, András Oláh, David Onchonga, Sahar Hammoud, Faten Amer, Viktória Prémusz, & Annamária Pakai.

## ETHICAL APPROVAL

Research ethics committee approval was obtained before research implementation both from the Scientific Research Committee at the Jordanian Ministry of Health (reg. # 21114), and the Ethics Committee at King Abdullah University Hospital (reg. # 13‐3‐17).

## PATIENT CONSENT FORM

No patients were involved in this study. However, the participants were asked to sign an informed consent form.

## Supporting information

Appendix S1

## Data Availability

The raw data that support the results of this research are available from the corresponding author upon a reasonable request.
